# ST8 and ST72 Methicillin-Resistant *S. aureus* Bacteremia in Korea: A Comparative Analysis of Clinical and Microbiological Characteristics

**DOI:** 10.3390/microorganisms13102399

**Published:** 2025-10-20

**Authors:** Yun Woo Lee, Ji-Hun Kim, So Yun Lim, Euijin Chang, Seongman Bae, Jiwon Jung, Min Jae Kim, Yong Pil Chong, Sung-Han Kim, Sang-Ho Choi, Sang-Oh Lee, Yang Soo Kim

**Affiliations:** 1 Division of Infectious Diseases, Department of Internal Medicine, Hallym University Sacred Heart Hospital, Anyang 14068, Republic of Korea; leeyunwoo@hallym.ac.kr; 2 Department of Medicine, Graduate School, University of Ulsan College of Medicine, Seoul 05505, Republic of Korea; 3 Department of Emergency Medicine, Uijeongbu St. Mary’s Hospital, The Catholic University of Korea, Uijeongbu 11765, Republic of Korea; remind1580@naver.com; 4 Division of Infectious Diseases, Asan Medical Center, University of Ulsan College of Medicine, Seoul 05505, Republic of Korea; soyun89lim@gmail.com (S.Y.L.); xenkins0618@gmail.com (E.C.); songman.b@gmail.com (S.B.); trueblue27@naver.com (J.J.); nahani99@gmail.com (M.J.K.); drchong@amc.seoul.kr (Y.P.C.); kimsunghanmd@hotmail.com (S.-H.K.); sangho@amc.seoul.kr (S.-H.C.); soleemd@amc.seoul.kr (S.-O.L.); 5 Center for Antimicrobial Resistance and Microbial Genetics, University of Ulsan College of Medicine, Seoul 05505, Republic of Korea

**Keywords:** *Staphylococcus aureus*, Methicillin-resistant *Staphylococcus aureus*, ST72, ST8/USA300, bacteremia, clinical outcome

## Abstract

Methicillin-resistant *Staphylococcus aureus* (MRSA) remains a major cause of bloodstream infection worldwide. In Korea, sequence type (ST) 72 has predominated, whereas ST8, including the USA300 lineage, has recently emerged. Comparative data on these genotypes in MRSA bacteremia (MRSAB) are limited. We conducted a retrospective cohort study of adult patients with MRSAB admitted to a 2700-bed tertiary care hospital in Republic of Korea between July 2008 and December 2020. Clinical features and outcomes of patients with ST8 MRSA were compared with those of patients with ST72 MRSA. Among 1975 cases of *S. aureus* bacteremia, 998 (50.5%) were due to MRSA, including 327 (32.7%) ST72 and 23 (2.3%) ST8 isolates. Demographics and comorbidities were similar, though pneumonia appeared more frequent in ST8 cases without statistical significance. ST8 isolates exhibited greater resistance to ciprofloxacin and erythromycin and more frequent vancomycin E-test MICs ≥1 mg/L, while broth microdilution MICs were comparable. *spa* type distribution differed, with t324 predominating in ST72 and t008 in ST8. Management practices, persistent bacteremia, recurrence, and 30- and 90-day mortality did not differ significantly. In multivariable analysis, liver cirrhosis and Charlson comorbidity index >4, but not MRSA genotype, independently predicted 30-day mortality. These findings highlight the importance of continued surveillance of emerging ST8 clones.

## 1. Introduction

*Staphylococcus aureus* remains a major human pathogen responsible for a broad spectrum of community- and healthcare-associated infections worldwide [1]. Since the introduction of antibiotics, the organism has acquired resistance to multiple agents, and methicillin-resistant *S. aureus* (MRSA) has emerged as a major healthcare problem [2,3]. MRSA initially emerged as a predominant cause of hospital- and healthcare-associated infections [1,4]. In the late 1990s, however, community-associated MRSA (CA-MRSA) clones became widespread, with sequence type (ST) 8-Staphylococcal Cassette Chromosome (SCC)*mec* IVa (USA300) rapidly establishing itself as the predominant lineage in the United States and subsequently spreading globally [5,6,7]. USA300 strains are typically Panton–Valentine leucocidin (PVL)-positive and have been strongly associated with skin and soft tissue infections, but they are also capable of causing severe invasive infections such as necrotizing pneumonia, osteomyelitis, septic arthritis, and bacteremia [7].

In contrast, the epidemiology of MRSA in Republic of Korea has been distinct. PVL-negative ST72-SCC*mec* IVc has been the predominant CA-MRSA clone in both community and healthcare settings [8,9,10]. Recently, however, ST8 lineages including USA300 have begun to emerge in Korea. Sporadic cases of USA300 infection were first reported in 2008 in a patient with a travel history to Hawaii, followed by additional cases without overseas exposure, implying in-country acquisition [11,12,13,14]. A multicenter Korean study identified PVL-positive ST8-MRSA (USA300) accounting for 0.4–12% of MRSA bacteremia across three centers, a range higher than expected [15]. Longitudinal surveillance has further demonstrated a steady increase in ST8 isolates, raising concerns about their potential expansion in both community and hospital settings [8]. Similar trends have been documented in neighboring countries such as Japan, where a distinct ST8 clone (ST8 CA-MRSA/J) with close genetic similarity to USA300 but unique virulence gene content has emerged and caused severe invasive infections [16]. In Taiwan as well, recent studies have reported a rising prevalence of USA300-like clones, underscoring the need for ongoing regional and global surveillance of these lineages [17].

Despite these observations, comparative studies of ST8 and ST72 in invasive infections remain scarce in Korea. In this study, we evaluated the clinical and microbiological characteristics and outcomes of MRSA bacteremia caused by ST8 and compared them with those caused by ST72, the long-dominant community genotype in Korea.

## 2. Materials and Methods

### 2.1. Study Design and Population

This retrospective cohort study was conducted using data from a previously established institutional cohort of adult patients with *S. aureus* bacteremia (SAB) admitted to a 2700-bed tertiary care hospital (Asan Medical Center, Seoul, Republic of Korea) between July 2008 and December 2020. All consecutive patients with a first episode of SAB were eligible. The exclusion criteria were as follows: (i) age under 18 years, (ii) discharge before culture results were available, which precluded outcome assessment, (iii) polymicrobial bacteremia, (iv) a previous episode of SAB within the preceding 90 days, (v) receipt of ≥3 days of treatment at another hospital, (vi) clinically insignificant bacteremia, defined as a single positive blood culture without signs or symptoms of infection that resolved without anti-staphylococcal therapy, and (vii) identification in outpatient settings. Clinical data, demographics, main focus of infection, and test results of metastatic infection, were collected within one week of the initial positive culture. All SAB cases were then reviewed by infectious disease specialists. Outcomes, including duration of bacteremia, recurrence, and death, were assessed through medical record review up to 90 days after the first positive culture. This study specifically analyzed and compared the clinical and microbiological characteristics and outcomes MRSA bacteremia caused by ST8 and ST72 isolates within this institutional cohort.

### 2.2. Laboratory and Microbiological Data

All *S. aureus* isolates were identified using the standard methods recommended by the Clinical and Laboratory Standards Institute (CLSI) and confirmed by morphological and biochemical testing, including the slide coagulase assay, or by matrix-assisted laser desorption ionization–time of flight (MALDI-TOF) mass spectrometry [18]. Antimicrobial susceptibility testing was interpreted according to standard criteria using the MicroScan system (Beckman Coulter, Brea, CA, USA) in accordance with the CLSI guidelines [18]. Methicillin resistance was determined based on oxacillin minimum inhibitory concentration (MIC) values and the detection of the *mec*A gene. Vancomycin MICs were assessed by both the broth microdilution (BMD) method and E test. BMD was performed following CLSI recommendations, whereas E test was performed according to the manufacturer’s protocol using vancomycin E test strips (bioMérieux, Marcy-l’Étoile, France). Multilocus sequence typing (MLST), assessment of δ-hemolysin activity for evaluation of accessory gene regulator (*agr*) function, and staphylococcal protein A (*spa*) genotyping were conducted as previously described [19,20,21]. These molecular analyses were performed on all MRSA isolates collected between July 2008 and December 2020.

### 2.3. Data Collection and Definitions

Electronic medical records were reviewed to collect data on age, sex, mode of acquisition, medical history, site of infection, severity at admission, microbiological results, and clinical outcomes for all enrolled patients. The mode of acquisition was categorized as community-acquired, community-onset healthcare-associated, or nosocomial, in accordance with the Friedman criteria [22]. The cause (main focus) of bacteremia was determined for each patient based on clinical, microbiological, and radiological findings, and classified as catheter-related, skin and soft tissue, bone and joint, pneumonia, endocarditis, urinary tract infection, arteriovenous graft infection, or primary bacteremia. Metastatic infection was defined as a newly developed sterile-site infection that was not clinically apparent at the time of the initial blood culture and was not identified during the initial diagnostic workup. The primary focus of infection was determined by infectious disease specialists based on microbiological evidence, radiological findings, and clinical course. The duration of bacteremia was defined as the interval between the first and last positive SAB culture, regardless of antimicrobial therapy. Recurrence was defined as the reappearance of signs and symptoms of infection more than 7 days after clinical improvement with confirmed negative blood cultures.

### 2.4. Statistical Analysis

Data manipulation and statistical analyses were conducted using R, version 4.0.4 (R Foundation for Statistical Computing, Vienna, Austria). Continuous variables were compared using the Wilcoxon rank-sum test, and categorical variables were analyzed using Fisher’s exact test or Pearson’s chi-squared test with simulated *p*-values, as appropriate. Univariate and multivariate logistic regression models were applied to identify independent risk factors for mortality. Multivariate models were adjusted for the following covariates: age ≥ 65, gender, hypertension, diabetes mellitus, liver cirrhosis, end-stage renal disease, chronic lung disease, solid cancer, hematologic malignancy, solid organ transplantation, ischemic heart disease, Charlson Comorbidity Index (CCI) > 4, focus of infection, presence of metastatic infection, and status of infection focus removal. To minimize small-sample bias due to the limited number of ST8 cases and zero 30-day deaths, Firth penalized and exact logistic regression analyses were additionally performed, including the prespecified covariates (ST type, age > 60, CCI > 4, and infection severity). Post hoc power for 30-day mortality difference was calculated (α = 0.05) with effect size expressed as Cohen’s *h*. Temporal trends in the annual proportions of ST72 and ST8 MRSA among SAB cases were analyzed using ANOVA, Kruskal–Wallis tests, and logistic regression with calendar year as a continuous variable. Yearly trends in the proportion of isolates with vancomycin MIC > 1 mg/L (by BMD and E-test) were also evaluated using binomial logistic regression, reporting odds ratios (ORs) per year with 95% confidence intervals. Because only 8 of 17 isolates in 2020 had available E-test results, MIC trend analyses were restricted to data up to 2019. To assess the potential clinical impact of vancomycin MIC, outcomes were further compared between isolates with MIC ≤1 mg/L and >1 mg/L. All reported *p*-values were two-sided, and a *p*-value < 0.05 was considered to indicate statistical significance.

## 3. Results

### 3.1. Baseline and Clinical Characteristics

A total of 1975 cases of SAB were identified during the study period, including 998 cases of MRSA (50.5%). ST72 and ST8 accounted for 327 (32.7%) and 23 (2.3%) of the MRSA isolates, respectively. The baseline characteristics of 350 patients with ST72 MRSA and ST8 MRSA are summarized in Table 1. Patients in both groups were of similar age (median, 64 years) and sex distribution, and the mode of acquisition did not differ significantly, with the majority of infections being healthcare-associated or hospital-acquired. The prevalence of comorbidities such as hypertension, diabetes mellitus, and solid cancer was generally comparable between the two groups. CCI scores and predisposing conditions, including recent surgery, prior antibiotic treatment, and use of immunosuppressive agents, were also similar across groups. With respect to the focus of infection, pneumonia was more common in the ST8 group (30% vs. 9.2%), although the difference did not reach statistical significance. Other infection sites, including catheter-related infections, bone and joint infection, skin and soft tissue infection, and endocarditis, were distributed similarly. The occurrence of metastatic infection (18% vs. 22%) and infection severity (sepsis or septic shock) did not differ significantly between ST72 and ST8 MRSA bacteremia.

### 3.2. Microbiological Characteristics

The microbiological characteristics and antibiotic susceptibilities of ST72 and ST8 MRSA isolates are summarized in Table 2. Overall, resistance to most antibiotics was comparable between the two groups; however, ST8 isolates demonstrated significantly higher resistance to ciprofloxacin (78% vs. 8.9%, *p* < 0.001) and erythromycin (61% vs. 26%, *p* = 0.003) compared with ST72 isolates. Resistance to clindamycin (13–20%) and gentamicin (13–30%) was observed at moderate frequencies, while resistance to fusidic acid, rifampin, and tetracycline remained low (<5%), with no significant differences between ST72 and ST8 isolates. All MRSA isolates were susceptible to vancomycin according to CLSI criteria; no vancomycin-resistant strains were identified. Vancomycin MICs determined by BMD were similar between the two groups, with the majority of isolates showing an MIC of 1 mg/L. However, E-test results revealed a significantly different distribution (*p* < 0.001), with ST8 isolates more frequently exhibiting MIC values ≥1 mg/L. Four ST72 isolates (1.2%) showed E-test MICs of 3 mg/L, which may reflect inter-method variability, as all values remained well below the CLSI resistance breakpoint. The prevalence of *agr* dysfunction did not differ significantly between ST72 and ST8 (12% vs. 17%, *p* = 0.501). *spa* typing revealed distinct clonal distributions: ST72 isolates were dominated by t324 (48%), t148 (12%), and diverse other types, whereas ST8 isolates were largely associated with *spa* type t008 (65%).

Over the 12-year study period, the annual proportion of ST8 MRSA among SAB cases gradually increased from 1.3% in 2008 to 3.3% in 2020, whereas ST72 remained relatively stable (Figure S1, Table S3). When analyzed by 4-year intervals (2008–2012, 2013–2016, 2017–2020), the ST8 proportion showed a significant upward trend (ANOVA *p* = 0.02; Kruskal–Wallis *p* = 0.04), while no significant change was observed for ST72. Logistic regression demonstrated a significant year-on-year increase for ST8 (OR 1.30 per year, *p* < 0.001) but not for ST72 (OR 1.03, *p* = 0.06) (Figure S2, Table S4). Logistic regression demonstrated a decreasing proportion of isolates with MIC > 1 mg/L for both methods (BMD: OR = 0.82 per year, 95% CI 0.73–0.91, *p* < 0.001; E-test: OR = 0.86 per year, 95% CI 0.79–0.93, *p* < 0.001; Figure S3).

Clinical outcomes did not differ significantly according to vancomycin MIC category (≤1 vs. >1 mg/L) (Table S5).

### 3.3. Clinical Course and Outcome

Management and clinical outcomes of patients with MRSA bacteremia are summarized in Table 3. Most patients in both groups received vancomycin with similar rates of therapeutic drug monitoring and comparable trough levels. The use of alternative agents, time to appropriate therapy, and duration of intravenous antibiotics did not differ significantly. The frequencies of eradicable foci, focus removal, and persistent bacteremia were also similar. Clinical outcomes, including 30- and 90-day mortality, showed no significant differences between ST72 and ST8 cases. The 30-day mortality rate was 13% in the ST72 group and 0% in the ST8 group. However, due to complete separation arising from the absence of deaths in the ST8 group, ORs could not be estimated. Fisher’s exact test revealed no statistically significant difference between the two groups (*p* = 0.093).

Univariable and multivariable logistic regression analyses were performed to identify risk factors associated with 30-day mortality (Table 4). In the univariable analysis, liver cirrhosis (OR, 3.26; 95% CI, 1.52–6.73; *p* = 0.002), solid cancer (OR, 3.03; 95% CI, 1.58–5.93; *p* < 0.001), and a CCI > 4 (OR, 4.70; 95% CI, 2.44–9.21; *p* < 0.001) were significantly associated with increased mortality. The presence of a removable focus was protective, with a significantly lower risk of 30-day mortality when the focus was removed (OR, 0.43; 95% CI, 0.20–0.86; *p* = 0.021).

In the multivariable analysis, liver cirrhosis (aOR, 2.82; 95% CI, 1.20–6.44; *p* = 0.015) and CCI > 4 (aOR, 4.67; 95% CI, 2.00–11.5; *p* < 0.001) remained independent predictors of 30-day mortality. Removal of the infectious focus showed a trend toward reduced mortality but did not reach statistical significance (aOR, 0.52; 95% CI, 0.23–1.11; *p* = 0.10). Other factors, including age, sex, MRSA genotype (ST72 vs. ST8), *agr* dysfunction, and vancomycin MIC, were not significantly associated with 30-day mortality.

In Firth and exact logistic regression analyses, the OR for 30-day mortality in ST8 versus ST72 was 0.17 (95% CI, 0.00–1.34; *p* = 0.11), consistent across both models (Table S1). In addition, a post hoc power analysis demonstrated that the study had sufficient statistical power (0.958; Cohen’s *h* = 0.795, α = 0.05) to detect the observed absolute difference in 30-day mortality between the groups, suggesting that the non-significant results were less likely due to limited sample size (Table S2).

In both univariate and multivariable analyses, ST8 MRSA was not significantly associated with 90-day recurrence or mortality compared with ST72 MRSA (Table 5).

## 4. Discussion

In this study, we compared the clinical and microbiological characteristics and outcomes of MRSA bacteremia caused by ST72 and ST8 isolates in Korea.

Baseline characteristics, including age, sex, comorbidities, and infection sites, were largely comparable between the two groups, which may partly explain the absence of significant differences in outcomes. Notably, pneumonia was more frequently observed among ST8 cases, echoing prior reports linking USA300-related clones with pulmonary infections [13,23]; however, in our cohort this trend did not reach statistical significance.

Microbiological analyses revealed distinct differences between the two clonal groups. ST8 isolates exhibited significantly higher resistance rates to ciprofloxacin and erythromycin and showed a predominance of *spa* type t008, consistent with the USA300 lineage described in other regions [5]. In contrast, ST72 isolates demonstrated more diverse *spa* types and lower resistance profiles. Although vancomycin MICs determined by BMD were similar between the groups, E-test results showed higher MIC distributions in ST8 isolates (mostly 1 mg/L). No vancomycin-resistant MRSA isolates (MIC ≥ 16 mg/L) were identified, and all values remained within the susceptible range by CLSI criteria [18]. This subtle upward shift underscores the need for continued surveillance of potential MIC creep [24,25]. However, this difference was not associated with adverse clinical outcomes in our study.

Despite these microbiological differences, management strategies and short-term outcomes did not differ significantly. Most patients in both groups received vancomycin therapy with therapeutic drug monitoring, and the use of alternative agents, duration of intravenous therapy, and frequency of source control procedures were comparable. Likewise, persistent bacteremia and 30- and 90-day mortality rates showed no significant differences, suggesting that clonal background exerted limited influence on treatment response or outcomes in this cohort. Nevertheless, ST8/USA300 typically harbors SCC*mec* IVa, arginine catabolic mobile element (ACME), and PVL genes, features that may confer enhanced virulence compared to other MRSA strains [26,27,28], whereas ST72-SCC*mec*IVc isolates are generally PVL-negative and their pathogenic potential remains less well defined [10,29]. Further genome-wide studies and functional analyses will be necessary to clarify the clinical implications of these clonal differences.

In this cohort, most MRSA bacteremia cases were healthcare-associated (40%) or hospital-acquired (49%), indicating that infections predominantly occurred in patients with prior healthcare exposure, such as those with vascular catheters, recent surgery, or indwelling devices. This pattern reflects the tertiary care hospital setting and aligns with known risk factors for MRSA bacteremia. Epidemiologically, ST72-SCC*mec*IVc has been the dominant community-associated MRSA clone in Korea since the mid-2000s but has increasingly spread into healthcare environments [10,29,30]. In contrast, ST8 MRSA, including USA300 and related variants, has emerged more recently. Although USA300 originally arose in the community in North America, recent Korean studies have identified its establishment within hospital settings, even among patients without travel history, suggesting local adaptation and circulation of hospital-associated ST8 lineages [15]. Continued genomic and epidemiological surveillance is warranted to differentiate imported USA300-like strains from locally evolved ST8 variants and to monitor their clinical and public health impact.

When risk factors for 30-day mortality were examined, host-related factors rather than bacterial genotype emerged as key determinants. Higher Charlson Comorbidity Index (CCI > 4) and underlying liver cirrhosis were independently associated with mortality, consistent with previous studies demonstrating the impact of comorbidity burden and cirrhosis on adverse outcomes in SAB [31,32]. Although the protective effect of source control did not remain statistically significant in the multivariable analysis, its clinical relevance is well supported in the literature [33,34]. Together, these findings suggest that in Korea, the prognosis of MRSA bacteremia is primarily driven by host comorbidities and adequacy of management, rather than clonal lineage.

Finally, analysis of 90-day outcomes demonstrated that ST8 was not significantly associated with increased recurrence or mortality compared with ST72. This further supports the notion that host factors, rather than clonal background, are the predominant determinants of prognosis in this setting. However, given the relatively small number of ST8 cases, these findings should be interpreted with caution, and larger multicenter studies are needed to validate them. Considering the established virulence and adverse clinical impact of USA300/ST8 in other regions [26,27,28], continued molecular epidemiologic surveillance remains essential to monitor potential shifts in clinical relevance.

This study expands upon our institution’s long-term SAB cohort, providing novel comparative insights into the clinical and microbiological distinctions between ST72 and ST8 MRSA clones—an aspect not specifically addressed in earlier analyses from the same cohort [8,10,29,32]. Through this work, we contribute further evidence to the understanding of MRSA clonal diversity and its clinical implications in Korea.

Our study had some limitations. First, it was conducted in a single center with a relatively small number of ST8 cases, which limits the generalizability of our findings and reduces the statistical power to detect subtle differences between clonal groups. In particular, the absence of 30-day mortality events in the ST8 group constrained statistical estimation, highlighting the challenges of interpreting rare outcomes in small cohorts. However, supplementary analyses using Firth and exact logistic regression, along with post hoc power estimation, demonstrated that our findings were robust and not substantially affected by small-sample bias. Second, the retrospective design introduces potential information bias and confounding, particularly regarding the assessment of clinical management such as adequacy of source control. In addition, the exclusion of patients diagnosed outside the hospital or transferred after more than three days may have introduced selection bias; however, these criteria were applied to ensure consistency in microbiological testing, antimicrobial stewardship, and clinical management within a single institutional framework. Third, detailed genotypic information such as PVL, ACME, and SCC*mec* subtypes was not available due to the retrospective nature of the study and limited sample availability. As a result, molecular confirmation of ST8 isolates as USA300 or locally evolved ST8 variants was not possible. The absence of these molecular data, together with the lack of virulence gene profiling or whole-genome sequencing, precluded a more comprehensive evaluation of clonal virulence characteristics and their potential impact on clinical outcomes. Further genomic characterization will be important to clarify the genetic relatedness of emerging ST8 strains in Korea and their potential epidemiologic significance. Therefore, our findings should be interpreted with caution, and larger, prospective, multicenter studies incorporating advanced molecular analyses are warranted to validate and extend these results.

## 5. Conclusions

In conclusion, clinical outcomes of MRSA bacteremia did not differ significantly between ST72 and ST8 isolates in Korea, despite distinct microbiological profiles. Mortality was mainly driven by host comorbidities such as liver cirrhosis and high CCI rather than clonal background. Although the small number of ST8 cases limits definitive interpretation, continued molecular surveillance is warranted to monitor the potential clinical impact of emerging ST8 clones.

## Figures and Tables

**Table 1:** Baseline and clinical characteristics.

Variable	ST72 (*n* = 327)	ST8 (*n* = 23)	Total (*n* = 350)	*p*
Age (years), median [IQR]	64 [53, 72]	64 [57, 74]	64 [54, 72]	0.323
Age > 60 years	197/327 (60%)	16/23 (70%)	213/350 (61%)	0.508
Male	198/327 (61%)	15/23 (65%)	213/350 (61%)	0.826
Mode of acquisition				0.783
Community-acquired	36/327 (11%)	3/23 (13%)	39/350 (11%)	
Healthcare-associated	130/327 (40%)	10/23 (43%)	140/350 (40%)	
Hospital-acquired	161/327 (49%)	10/23 (43%)	171/350 (49%)	
Comorbidities				
Hypertension	145/327 (44%)	14/23 (61%)	159/350 (45%)	0.135
Diabetes mellitus	119/327 (36%)	13/23 (57%)	132/350 (38%)	0.074
Liver cirrhosis	47/327 (14%)	2/23 (8.7%)	49/350 (14%)	0.754
End-stage renal disease	50/327 (15%)	5/23 (22%)	55/350 (16%)	0.381
Chronic lung disease ^a^	7/327 (2.1%)	0/23 (0%)	7/350 (2.0%)	>0.999
Solid cancer	123/327 (38%)	6/23 (26%)	129/350 (37%)	0.372
Hematologic malignancy	31/327 (9.5%)	1/23 (4.3%)	32/350 (9.1%)	0.708
Solid organ transplantation	14/327 (4.3%)	0/23 (0%)	14/350 (4.0%)	0.611
Ischemic heart disease	36/327 (11%)	0/23 (0%)	36/350 (10%)	0.149
CCI, median [IQR]	2 [2, 5]	2 [1, 4]	2 [2, 5]	0.136
CCI > 4	84/327 (26%)	5/23 (22%)	89/350 (25%)	0.807
Predisposing condition				
Recent surgery ^b^	53/327 (16%)	2/23 (8.7%)	55/350 (16%)	0.552
Prior antibiotic treatment ^b^	109/327 (33%)	4/23 (17%)	113/350 (32%)	0.165
Neutropenia	17/327 (5.2%)	0/23 (0%)	17/350 (4.9%)	0.615
Anticancer chemotherapy	60/327 (18%)	3/23 (13%)	63/350 (18%)	0.779
Immunosuppressive treatment ^b^	83/327 (25%)	2/23 (8.7%)	85/350 (24%)	0.081
Central venous catheter	96/327 (29%)	7/23 (30%)	103/350 (29%)	>0.999
Non-catheter indwelling devices	65/327 (20%)	8/23 (35%)	73/350 (21%)	0.109
Cardiac implantable electronic device	4/327 (1.2%)	2/23 (8.7%)	6/350 (1.7%)	0.053
Prosthetic heart valve	12/327 (3.7%)	0/23 (0%)	12/350 (3.4%)	>0.999
Vascular graft	36/327 (11%)	5/23 (22%)	41/350 (12%)	0.168
Orthopedic implant	14/327 (4.3%)	2/23 (8.7%)	16/350 (4.6%)	0.283
Others	9/327 (2.8%)	1/23 (4.3%)	10/350 (2.9%)	0.498
Main focus of infection				
Central catheter related	62/327 (19%)	2/23 (8.7%)	64/350 (18%)	0.160
Peripheral catheter related	23/327 (7.0%)	0/23 (0%)	23/350 (6.6%)	
Primary bacteremia (unidentified)	52/327 (16%)	4/23 (17%)	56/350 (16%)	
Pneumonia	30/327 (9.2%)	7/23 (30%)	37/350 (11%)	
Bone and joint infection	36/327 (11%)	2/23 (8.7%)	38/350 (11%)	
Surgical wound	20/327 (6.1%)	0/23 (0%)	20/350 (5.7%)	
Skin and soft tissue	28/327 (8.6%)	2/23 (8.7%)	30/350 (8.6%)	
Endocarditis	14/327 (4.3%)	0/23 (0%)	14/350 (4.0%)	
Urinary tract	8/327 (2.4%)	1/23 (4.3%)	9/350 (2.6%)	
Arteriovenous graft	17/327 (5.2%)	2/23 (8.7%)	19/350 (5.4%)	
Others	37/327 (11%)	3/23 (13%)	40/350 (11%)	
Metastatic infection	60/327 (18%)	5/23 (22%)	65/350 (19%)	0.781
Severity of infection				
No sepsis	66/326 (20%)	5/23 (22%)	71/349 (20%)	
Sepsis	225/326 (69%)	15/23 (65%)	240/349 (69%)	
Septic shock ^c^	35/326 (11%)	3/23 (13%)	38/349 (11%)	

Data are presented as the number of patients (%), unless otherwise indicated. ST, sequence type; IQR, interquartile range; CCI, Charlson Comorbidity Index. ^a^ Includes chronic pulmonary obstructive lung disease and bronchiectasis. ^b^ Within a month before MRSA bacteremia. ^c^ Sepsis with persistent hypotension that requires vasopressors to maintain mean arterial pressure ≥ 65 mmHg, and lactate level ≥ 2 mmol/L despite adequate fluid resuscitation.

**Table 2:** Antibiotic resistance profile and microbiological characteristics.

Characteristic	ST72 (*n* = 327)	ST8 (*n* = 23)	Total (*n* = 350)	*p*
Antibiotics resistance				
Clindamycin	65/327 (20%)	3/23 (13%)	68/350 (19%)	0.615
Ciprofloxacin	29/327 (8.9%)	18/23 (78%)	47/350 (13%)	<0.001
Erythromycin	84/327 (26%)	14/23 (61%)	98/350 (28%)	0.003
Fusidic acid	6/327 (1.8%)	1/23 (4.3%)	7/350 (2.0%)	0.564
Gentamicin	44/327 (13%)	7/23 (30%)	51/350 (15%)	0.111
Rifampin	8/327 (2.4%)	1/23 (4.3%)	9/350 (2.6%)	0.074
Trimethoprim/sulfamethoxazole	2/327 (0.6%)	0/23 (0%)	2/350 (0.6%)	>0.999
Tetracycline	9/327 (2.8%)	1/23 (4.3%)	10/350 (2.9%)	0.564
Vancomycin MIC by BMD method				0.527
0.5	5/327 (1.5%)	0/23 (0%)	5/350 (1.4%)	
1	281/327 (86%)	22/23 (96%)	303/350 (87%)	
2	41/327 (13%)	1/23 (4.3%)	42/350 (12%)	
Vancomycin MIC by E test method				<0.001
0.5	0 (0%)	2 (9.5%)	2 (0.6%)	
0.75	8 (2.5%)	2 (9.5%)	10 (2.9%)	
1	62 (19%)	12 (57%)	74 (22%)	
1.5	180 (56%)	3 (14%)	183 (54%)	
2	66 (21%)	2 (9.5%)	68 (20%)	
3	4 (1.3%)	0 (0%)	4 (1.2%)	
*agr* dysfunction	38/327 (12%)	4/23 (17%)	42/350 (12%)	0.501
*spa* types ^a^				<0.001
t008	1 (0.3%)	15 (65%)	16 (4.6%)	
t148	40 (12%)	0 (0%)	40 (11%)	
t324	157 (48%)	0 (0%)	157 (45%)	
t664	49 (15%)	0 (0%)	49 (14%)	
Unknown	26 (8.0%)	1 (4.3%)	27 (7.7%)	
others	54 (17%)	7 (30%)	61 (17%)	

Data are presented as the number of patients (%). ^a^ Only the five most frequent *spa* types are presented; all remaining *spa* types were grouped as “others”. ST, sequence type; MIC, minimum inhibitory concentration; BMD, broth microdilution method.

**Table 3:** Management and clinical outcomes of patients with MRSA bacteremia.

Outcome	ST72 (*n* = 327)	ST8 (*n* = 23)	Total (*n* = 350)	*p*
Management				
Transthoracic echocardiography	275/327 (84%)	20/23 (87%)	295/350 (84%)	>0.999
Transesophageal echocardiography	63/327 (19%)	5/23 (22%)	68/350 (19%)	0.786
Vancomycin therapy	277/327 (85%)	22/23 (96%)	299/350 (85%)	0.222
TDM performed	250/327 (76%)	19/23 (83%)	269/350 (77%)	0.615
Trough level, median (IQR), mg/L	11.5 [8.1, 18.7]	11.7 [8.9, 18.2]	11.5 [8.2, 18.4]	0.8
Antibiotics other than vancomycin				
Teicoplanin	62/327 (19%)	1/23 (4.3%)	63/350 (18%)	0.093
Linezolid	44/327 (13%)	1/23 (4.3%)	45/350 (13%)	0.334
Time to appropriate antimicrobials, days	1.00 [0.00, 2.00]	0.00 [0.00, 2.00]	1.00 [0.00, 2.00]	0.528
Duration of intravenous antimicrobials, days	20 [12, 32]	17 [13, 40]	19 [12, 33]	0.824
Removable focus	169 (52%)	8 (35%)	177 (51%)	0.13
Focus removed	148 (88%)	8 (100%)	156 (88%)	0.6
Length of bacteremia				
Bacteremia ≥ 3 days	130/327 (40%)	9/23 (39%)	139/350 (40%)	>0.999
Bacteremia ≥ 7 days	62/327 (19%)	6/23 (26%)	68/350 (19%)	0.667
30-day mortality	43/327 (13%)	0/23 (0%)	43/350 (12%)	0.093
90-day mortality	75/327 (23%)	4/23 (17%)	79/350 (23%)	0.796
90-day recurrence	18/327 (5.5%)	1/23 (4.3%)	19/350 (5.4%)	>0.999

Data are presented as the number of patients (%), unless otherwise indicated. ST, sequence type; TDM; therapeutic drug monitoring; IQR, interquartile range.

**Table 4:** Univariable and multivariable analysis of risk factors associated with 30-day mortality in 350 adult patients with MRSA bacteremia.

Risk Factor	Univariable Analysis		Multivariable Analysis ^a^	
	OR [95% CI]	*p*	OR [95% CI]	*p*
Age ≥ 65 years	1.30 [0.69, 2.49]	0.419	1.64 [0.81, 3.40]	0.2
Male	0.88 [0.46, 1.70]	0.697	0.68 [0.33, 1.42]	0.3
Hypertension	0.68 [0.35, 1.30]	0.250		
Diabetes mellitus	0.60 [0.29, 1.19]	0.160		
Liver cirrhosis	3.26 [1.52, 6.73]	0.002	2.82 [1.20, 6.44]	0.015
End-stage renal disease	0.23 [0.04, 0.79]	0.049	0.29 [0.04, 1.17]	0.13
Chronic lung disease ^b^	1.19 [0.06, 7.23]	0.871		
Solid cancer	3.03 [1.58, 5.93]	<0.001	0.93 [0.37, 2.25]	0.9
Hematologic malignancy	1.02 [0.29, 2.78]	0.969		
Solid organ transplantation	0.54 [0.03, 2.81]	0.556		
Ischemic heart disease	0.62 [0.15, 1.84]	0.450		
CCI > 4	4.70 [2.44, 9.21]	<0.001	4.67 [2.00, 11.5]	<0.001
Central catheter related	Reference	–		
Peripheral catheter related	1.05 [0.21, 4.04]	0.946		
Pneumonia	2.25 [0.78, 6.61]	0.132		
Bone and joint infection	0.19 [0.01, 1.09]	0.124		
Skin and soft tissue	0.78 [0.16, 2.93]	0.726		
Unknown (primary bacteremia)	1.34 [0.48, 3.83]	0.577		
Others	1.24 [0.38, 3.86]	0.717		
Metastatic infection	0.54 [0.18, 1.32]	0.217		
Removable focus				
No removable focus	Reference	–	Reference	–
Focus removed	0.43 [0.20, 0.86]	0.021	0.52 [0.23, 1.11]	0.10
Focus not removed	0.86 [0.19, 2.77]	0.823	1.29 [0.26, 4.68]	0.7
MRSA genotype				
ST72	Reference	–	Reference	–
ST8 ^c^	Not estimable	0.985	Not estimable	>0.9
*agr* dysfunction	0.96 [0.32, 2.40]	0.936		
Vancomycin MIC by BMD				
0.5	Reference	–		
1	0.36 [0.05, 7.34]	0.378		
2	2.00 [0.06, 73.7]	0.676		

OR, odds ratio; CI, confidence interval; CCI, Charlson Comorbidity Index; ST, sequence type; MIC, minimal inhibitory concentration; BMD, broth microdilution. ^a^ Multivariable analysis included age, sex, MRSA genotype and variables showing significant differences (*p* < 0.05) in the univariable analysis. ^b^ Includes chronic pulmonary obstructive lung disease and bronchiectasis. ^c^ Due to complete separation (0 deaths in the ST8 group), odds ratios for 30-day mortality could not be estimated; Fisher’s exact test showed no significant difference (*p* = 0.093).

**Table 5:** OR for recurrence and mortality at 90 days in patients with ST8 MRSA compared to those with ST72 MRSA.

Analyses	Recurrence		Mortality	
	OR (95% CI)	*p*	OR (95% CI)	*p*
Univariate	0.78 [0.04–4.06]	0.813	0.71 [0.2–1.95]	0.540
Multivariate	1.06 [0.05–7.22]	0.962	0.77 [0.19–2.43]	0.672

Multivariate analyses were adjusted for age ≥ 65 years, gender, comorbidities, Charlson comorbidity index ≥ 4, main focus of infection, metastatic infection, and removal of infection focus. OR, odds ratios; ST, sequence type; MRSA, methicillin-resistant Staphylococcus aureus; CI, confidence interval.

## Data Availability

The original contributions presented in the study are included in the article/Supplementary Materials, further inquiries can be directed to the corresponding author.

## Supplementary Materials

The following supporting information can be downloaded at: https://www.mdpi.com/article/10.3390/microorganisms13102399/s1, Figure S1. Yearly distribution of SAB, MRSA, ST72, and ST8 (2008–2020). Figure S2. Proportion of ST72, and ST8 across study periods (2008–2012, 2013–2016, 2017–2020). Figure S3. Proportion of isolates with MIC >1 mg/L by year. Table S1. Comparison of Firth and Exact Logistic Regression for 30-day Mortality (ST72 vs. ST8). Table S2. Post-hoc Power Analysis for 30-day Mortality (ST72 vs. ST8 MRSA bacteremia). Table S3. Yearly numbers and proportions of ST72 and ST8 among total *S. aureus* bacteremia. Table S4. Temporal trend analysis of ST72 and ST8 MRSA among *S. aureus bacteremia* cases (2008–2020). Table S5. Outcomes according to vancomycin MIC.

## Abbreviations

The following abbreviations are used in this manuscript:

MRSA	Methicillin-resistant *Staphylococcus aureus*
ST	Sequence type
CA-MRSA	Community-associated methicillin-resistant *Staphylococcus aureus*
PVL	Panton–Valentine leucocidin
SAB	*S. aureus* bacteremia
MIC	Minimum inhibitory concentration
BMD	Broth microdilution
MLST	Multilocus sequence typing
*agr*	accessory gene regulator
*spa*	staphylococcal protein A
CCI	Charlson Comorbidity Index
OR	Odds ratio
SCC	Staphylococcal Chromosomal Cassette
ACME	Arginine Catabolic Mobile Element
