# Supplementary material for: ST8 and ST72 Methicillin-Resistant S. aureus Bacteremia in Korea: A Comparative Analysis of Clinical and Microbiological Characteristics
Source: Microorganisms. 2025 Oct 20;13(10):2399. doi: 10.3390/microorganisms13102399 (PMC12566240; doi:10.3390/microorganisms13102399)

Table S1. Comparison of Firth and Exact Logistic Regression for 30-day Mortality (ST72 vs ST8).

	Firth penalized logistic regression			Exact logistic regression		
	**exp(β)**	**95% CI**	**p-value**	**exp(β)**	**95% CI**	**p-value**
MRSA genotype						
ST72	–	–	–	–	–	–
ST8	0.17	0.00–1.34	0.11	0.17	0.00–1.34	0.11
Age > 60 years	1.13	0.55– 2.38	0.7	1.13	0.55–2.38	0.7
CCI > 4	4.76	2.32–9.95	<0.001	4.76	2.32–9.95	<0.001
Severity of infection						
No sepsis	–	–	–	–	–	–
Sepsis	2.59	0.91–10.0	0.078	2.59	0.91–10.0	0.078
Septic shock	4.87	1.15–23.4	0.031	4.87	1.15–23.4	0.031

OR, odds ratio; CI, confidence interval; ST, sequence type; CCI, Charlson Comorbidity Index

Table S2. Post-hoc Power Analysis for 30-day Mortality (ST72 vs. ST8 MRSA bacteremia).

**Metric**	**Value**
Post-hoc power	0.958
Effect size (Cohen's *h*)	0.795
n1 (ST72)	316
n2 (ST8)	23
Significance level (α)	0.05

Table S3. Yearly numbers and proportions of ST72 and ST8 among total *S. aureus* bacteremia.

Year	Total SAB	MRSA	ST72	ST8	ST72(%)	ST8(%)
2008	77	42	8	1	10.4	1.3
2009	221	131	28	0	12.7	0.0
2010	204	124	34	1	16.7	0.5
2011	223	138	32	0	14.3	0.0
2012	77	42	15	1	19.5	1.3
2013	165	70	27	0	16.4	0.0
2014	192	94	37	1	19.3	0.5
2015	169	71	28	0	16.6	0.0
2016	147	74	28	4	19.0	2.7
2017	159	69	29	3	18.2	1.9
2018	147	69	35	5	23.8	3.4
2019	103	34	12	2	11.7	1.9
2020	91	40	14	3	15.4	3.3

SAB, *S. aureus* bacteremia; MRSA, Methicillin-resistant *S. aureus*; ST, sequence type

Table S4. Temporal trend analysis of ST72 and ST8 MRSA among *S. aureus bacteremia* cases (2008–2020).

**Clone**	**Period (Mean %)**			**ANOVA p**	**Kruskal p**	**OR per year**	**p value**
	**2008–2012**	**2013–2016**	**2017–2020**				
ST72	14.7	17.8	17.3	0.43	0.53	1.03	0.063
ST8	0.6	0.8	2.6	0.02	0.04	1.30	0.0004

Mean proportions were calculated for each 4-year period.
Between-period differences were assessed by ANOVA and Kruskal–Wallis tests.

Table S5. Outcomes according to vancomycin MIC.

Outcome	MIC ≤1 mg/L (n = 86)	MIC >1 mg/L (n = 255)	p-value
ICU care, n (%)	4 (4.7)	17 (6.7)	0.611
Median length of bacteremia (days) [IQR]	2 [1–8]	1 [1–4]	0.056
Bacteremia ≥7 days, n (%)	23 (27)	43 (17)	0.044
Bacteremia ≥3 days, n (%)	37 (43)	98 (38)	0.337
30-day mortality, n (%)	12 (14)	30 (12)	0.574
90-day mortality, n (%)	21 (24)	57 (22)	0.767
90-day recurrence, n (%)	3 (3.5)	16 (6.3)	0.423

IQR, interquartile range; MIC, minimum inhibitory concentration.

Supplementary Figure S1. Yearly distribution of SAB, MRSA, ST72, and ST8 (2008-2020).

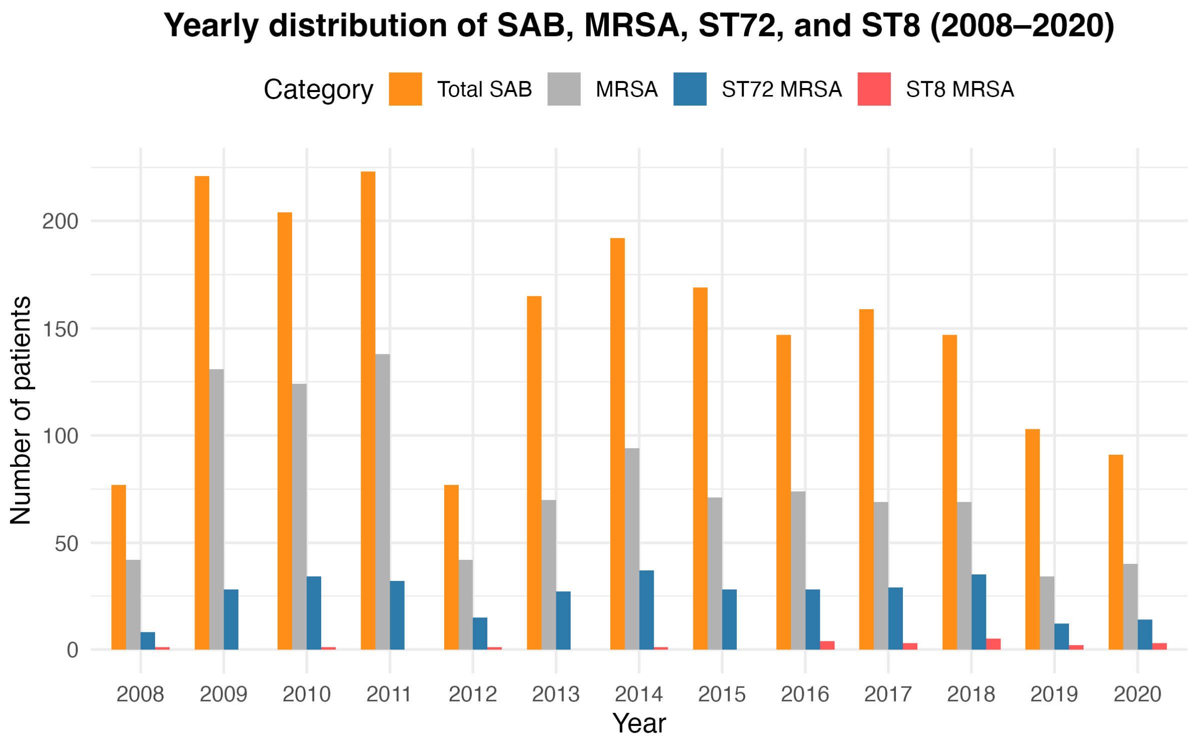

Supplementary Figure S2. Proportion of ST72, and ST8 across study periods (2008-2012, 2013-2016, 2017-2020).

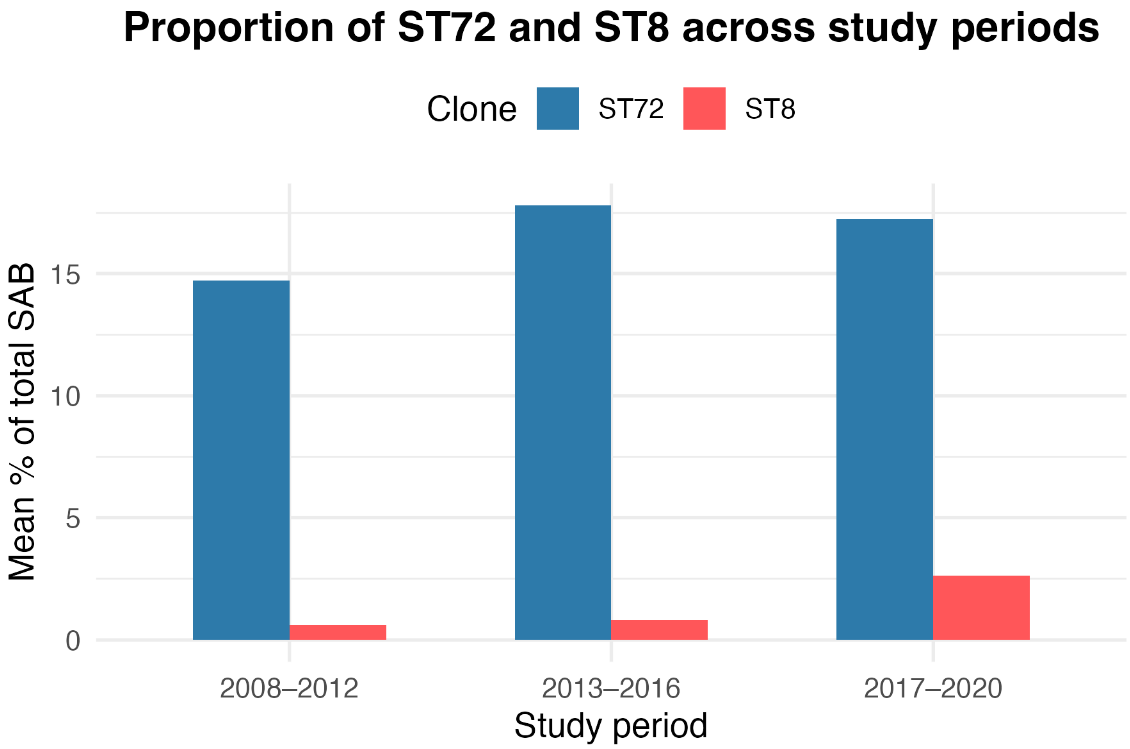

Supplementary Figure S3. Proportion of isolates with MIC >1mg/L by year.

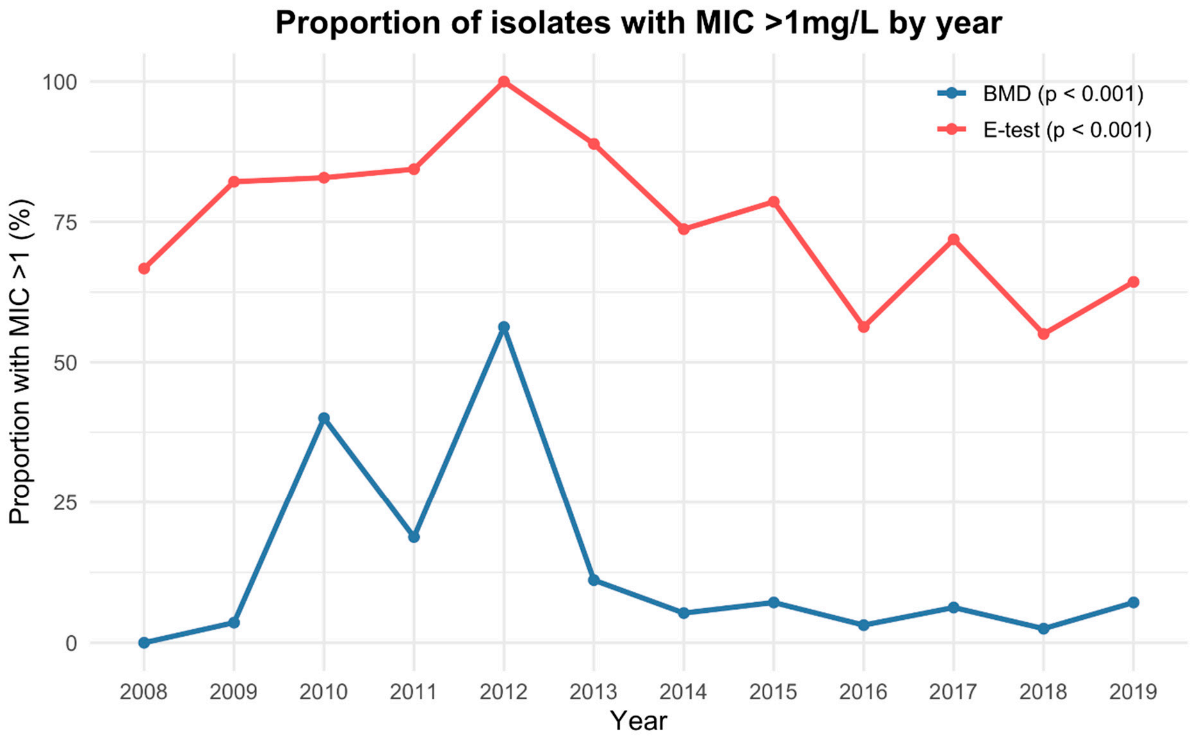

Supplement: Supplementary file 1 [file microorganisms-13-02399-s001.zip › microorganisms-3922770-supplementary.pdf]
